# A sequence variant in the diacylglycerol *O*-acyltransferase 2 gene influences palmitoleic acid content in pig muscle

**DOI:** 10.1038/s41598-021-94235-z

**Published:** 2021-07-20

**Authors:** Emma Solé, Roger Ros-Freixedes, Marc Tor, Ramona N. Pena, Joan Estany

**Affiliations:** Department of Animal Science, University of Lleida-Agrotecnio-CERCA Center, 191 Rovira Roure, 25198 Lleida, Catalonia Spain

**Keywords:** Genetics, Zoology, Biomarkers

## Abstract

The bulk of body fat in mammals is in the form of triacylglycerol. Diacylglycerol *O*-acyltransferase 2 (DGAT2) catalyses the terminal step in triacylglycerol synthesis. The proximity of DGAT2 with stearoyl-CoA desaturase (SCD) in the endoplasmic reticulum may facilitate provision of de novo SCD-mediated fatty acids as substrate for DGAT2. Here, we first searched for sequence variants in the *DGAT2* gene to then validate their effect on fat content and fatty acid composition in muscle, subcutaneous fat and liver of 1129 Duroc pigs. A single nucleotide polymorphism in exon 9 (ss7315407085 G > A) was selected as a tag variant for the 33 sequence variants identified in the *DGAT2* region. The *DGAT2*-G allele increased *DGAT2* expression in muscle and had a positive impact on muscular C14 and C16 fatty acids at the expense of C18 fatty acids. Although there was no evidence for an interaction of *DGAT2* with functional *SCD* genotypes, pigs carrying the *DGAT2*-G allele had proportionally more palmitoleic acid relative to palmitic acid. Our findings indicate that DGAT2 preferentially uptakes shorter rather than longer-chain fatty acids as substrate, especially if they are monounsaturated, and confirm that fatty acid metabolism in pigs is subjected to subtle tissue-specific genetic regulatory mechanisms.

## Introduction

Meat fat content and composition are important attributes that contribute to the nutritional quality and the consumer perception of meat products. Total fat dietary intake, particularly intake of saturated fatty acid (SFA), has been associated with obesity, circulating LDL-cholesterol and increased risk of coronary heart disease^1^. In line with this, public health recommendations advocate for low-fat diets and dietary substitution of SFA with monounsaturated fatty acids (MUFA) and polyunsaturated fatty acids (PUFA)^2–5^. On the other hand, although most consumers already prefer leaner meat products^6^, intramuscular fat (IMF) content and fatty acid composition exert a great influence on meat organoleptic and technological properties^7,8^. Increasing the knowledge of the genetic basis of the regulation of fat metabolism is key to improve the quality of meat products.

**Figure 1:**
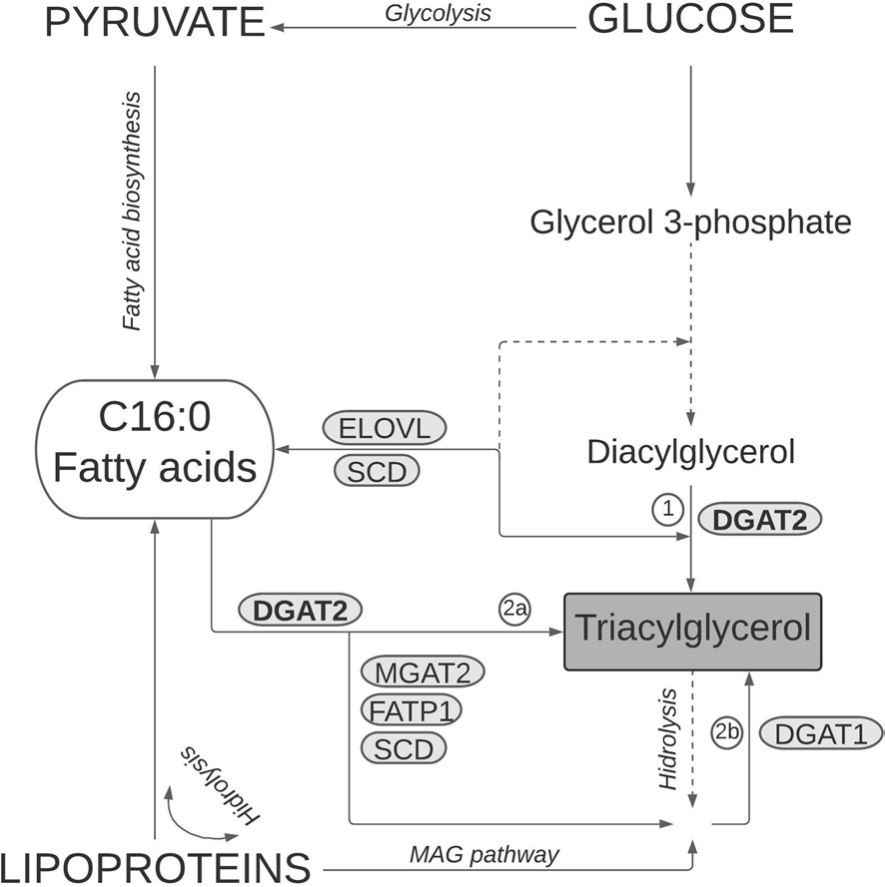
The role of DGAT2 in the synthesis pathway of triacylglycerol. Triacylglycerol is (1) synthesized de novo by sequential addition of fatty acyl moieties to a glycerol 3-phosphate (the G3P pathway) or (2) formed through the re-esterification of partial glycerides that results from either the hidrolysis of pre-existing triacylglycerol or via the monoacylglycerol (MAG) pathway. De novo transformation of diacylglycerol to triacylglycerol is catalyzed by DGAT2 (1) but re-esterification of partial glycerides is done by either DGAT2 (2a) or DGAT1 (2b). However, while DGAT1 specifically incorporates pre-formed fatty acids, DGAT2, which is able to form protein complexes with MGAT2, FATP1 and SCD, can use both endogenous and pre-formed fatty acids as substrates. *DGAT1* diacylglycerol *O*-acyltransferase 1, *DGAT2* diacylglycerol *O*-acyltransferase 2, *ELOVL* fatty acid elongase, *FATP1* fatty acid transport protein 1, *MGAT2* monoacylglycerol acyltransferase 2, *SCD* stearoyl-CoA desaturase.

Triacylglycerols (TG) are the main stores of metabolic energy, serving as a reservoir of essential and non-essential fatty acids, and the precursors of phospholipids. In mammals, TG are primarily synthesized in the liver, intestine and adipose tissue^9^. Diacylglycerol *O*-acyltransferase (*DGAT1* and *DGAT2*) genes are involved in intestinal fat absorption and play a critical role in the synthesis of TG in the adipocytes^10,11^. In particular, *DGAT1* and *DGAT2* encode membrane proteins that catalyse the formation of the ester bond between the hydroxyl group of 1,2-diacylglycerol and a long-chain fatty acyl-CoA (Fig. 1)^9,12,13^, the final step in the production of TG in mammals^12–15^. Although DGAT1 and DGAT2 have similar functions in the adipocytes, DGAT2 has been found to be more critical in mice, where it is essential for post-natal survival and can as well compensate for disruptions of DGAT1^16–18^. Recent findings indicate that DGAT2 is specifically engaged in de novo lipogenesis by favouring both the synthesis of TG from glycerol 3-phosphate and the incorporation of endogenous fatty acids into TG, particularly the MUFA that result from the desaturation of palmitate (C16:0) and stearate (C18:0) through the action of stearoyl-CoA desaturase (SCD)^19^. DGAT2 and SCD can localize in the endoplasmic reticulum membrane and this proximity may facilitate the provision of de novo SCD-mediated MUFA as substrate for DGAT2 to form TG.

Polymorphisms within *DGAT2* have the potential of affecting lipogenesis. Thus, *DGAT2* has been investigated as a candidate gene for fat-related traits such as obesity in humans^11,20^ and for lean content and muscle and backfat fatty acid composition in livestock species, particularly in pigs^14,21,22^. As a result of a genome-wide association study (GWAS) for fatty acid composition in Duroc pigs, we found that the region on SSC9 where *DGAT2* lies was associated with MUFA content. Here, using this Duroc line as a model, we investigate the role of *DGAT2* variants on fat metabolism in pigs. We first searched for sequence variants in the promoter, coding, and 3′-untranslated regions of porcine *DGAT2* and then validated their effect on fat content and composition using data and samples from a large biorepository^23^. Given that this line segregates for a functional variant in the *SCD* gene^24^, potential interactions of *DGAT2* with *SCD* were also assessed.

## Materials and methods

### Ethics statement

Pigs used in the study were raised and slaughtered in commercial units following applicable regulations and good practice guidelines on the protection of animals kept for farming purposes, during transport and slaughter (Royal Decree 37/2014, Spain) and tissue samples used were collected from these pigs at the slaughterhouse. In agreement with the European directive 2010/63/UE, tissue sampling from non-experimental animals slaughtered following legal procedures is not under the scope of European regulation on animal experimentation and does not require specific project authorization. The experimental protocol was approved by the Ethical Committee on Animal Experimentation of the University of Lleida. The study was carried out in compliance with the ARRIVE guidelines.

### Animals and phenotypes

A total of 1129 pigs from 158 sires and 559 dams of the same Duroc line were used in the experiment. Pigs were raised in 15 fattening batches between 2006 and 2016 following a similar protocol for data recording and tissue sampling^25^. In each batch, pigs were raised from 75 days of age until slaughter in the same farm under identical conditions. During this period, pigs had ad libitum access to commercial feed (Esporc, Riudarenes, Girona, Spain). At 206 (SD 8) days of age, all pigs were weighted and backfat (BT) and loin (LT) thickness were ultrasonically measured at 5 cm off the midline between the third and fourth last ribs using the portable equipment Piglog 105 (Frontmatec, Kolding, Denmark). Pigs were slaughtered at 211 (SD 9) days in a commercial slaughterhouse equipped with a carbon dioxide stunning system. Carcass composition traits were recorded, including carcass weight, BT and LT. Both BT and LT were measured at 6 cm off the midline between the third and fourth last ribs using an ultrasound automatic scanner (AutoFOM, SFK-Technology, Denmark). Immediately after slaughter, a sample of *semimembranosus* muscle (SM, *n* = 40) and subcutaneous fat (SF, *n* = 226) were collected, snap-frozen and stored at -80ºC. After chilling for about 24 h at 2 °C, a large sample of the muscles *gluteus medius* (GM, *n* = 1093) and *longissimus thoracis* (LM, *n* = 526) were collected, vacuum packaged, and stored at − 20 °C until required. The IMF content in GM and LM, as well as the fatty acid composition in GM, LM and SF were determined in duplicate by quantitative gas chromatography^26^. The amount of each fatty acid was expressed as the percentage of each individual fatty acid relative to total fatty acid. The proportion of SFA (C14:0, C16:0, C18:0 and C20:0), MUFA (C16:1n-7, C18:1n-9, C18:1n-7 and C20:1n-9) and PUFA (C18:2n-6, C18:3n-3, C20:2n-6 and C20:4n-6) were calculated.

### Genome-wide association study

In order to identify candidate genomic regions associated with fat-related traits, GWAS for each individual fatty acid were performed using a subset of 254 pigs. The genomic DNA of these pigs was isolated as described in Ref.^24^ and used for single nucleotide polymorphism (SNP) genotyping with either the PorcineSNP60 v2 Genotyping BeadChip (*n* = 138) or the GGP Porcine HD Array (*n* = 116) porcine arrays (Illumina, San Diego, CA, USA). Those SNPs that displayed a minor allele frequency below 0.10, a call rate below 0.95, or that could not be mapped to *Sus scrofa* reference genome (Sscrofa11.1)^27^ were filtered out. A total of 36,000 SNPs remained after data quality control. For each trait, a GWAS was performed by fitting a linear mixed model using GEMMA^28^, where phenotypes were adjusted for batch (11 levels) and IMF content as a covariate. The association of each SNP was tested using the Wald statistic considering the Bonferroni correction for multiple testing. Significance was set to a level of P ≤ 1.4 × 10^–6^. Two regions were found to be associated with C16:1n-7, one on SSC9 and another on SSC14, the latter corresponding to a reported polymorphism in the *SCD* gene that is known to affect MUFA and particularly C16:1n-7^24^. Candidate genes mapping within the SSC9 region were explored with Ensembl Genes Database using BioMart (https://www.ensembl.org/biomart/martview). Functional analyses like Gene Ontology and Reactome Pathway Enrichment Analysis were performed using Enrichr^29^. *DGAT2* was retrieved as the most promising candidate gene for C16:1n-7 on SSC9.

### Sequence variation in *DGAT2*

Variant discovery in porcine *DGAT2* was examined by retrieving all sequence variants from the coding region, the 3′-untranslated region and 500 bp upstream on the proximal promoter of the gene (SSC9 10,031,627 to 10,068,464 bp) in a subset of 199 pigs of the same line with whole-genome sequencing data available. Sequenced pigs covered all representative sire families used in the experiment. DNA samples were submitted to Centre Nacional d’Anàlisi Genòmica (CNAG-CRG, Barcelona, Spain) for sequencing. Libraries were prepared and sequenced with paired-end reads with a NovaSeq 6000 instrument (Illumina) according to the manufacturer’s protocol. Libraries were aligned to the Sscrofa11.1^27^ using the BWA-MEM algorithm^30^. The average realized sequencing coverage was 6.8x (SD = 1.2×; min = 4.4×; max = 12.2×). Variants were identified following GATK HaplotypeCaller 3.8.0 software^31,32^. The SNP ss7315407085 G > A in exon 9 (SNP5, 9:10,065,826; Table 1) was selected for further validation with the whole set of pigs.

**Table 1:** Position, alelles and location of the variants identified in the genomic region from SSC9 10,031,627 to 10,068,464 bp, which includes coding region, 3′-untranslated region and 500 bp upstream on the proximal promoter of the *DGAT2* gene.

Variant	Position (bp)	Location	*n*^a^	MAF^b^	Major/minor alleles
SNP1	10,031,901	Promoter	30	0.35	C/A
SNP2	10,032,155	Promoter	97	0.01	T/C
SNP3	10,054,197	Exon 3	183	0.09	C/T
SNP4	10,063,315	Exon 7	179	0.08	A/G
SNP5	10,065,826	Exon 9	120	0.28	G/A
SNP6	10,065,866	3′-UTR	119	0.23	G/A
SNP7	10,066,289	3′-UTR	138	0.49	G/C
SNP8	10,066,329	3′-UTR	142	0.30	C/T
SNP9	10,066,334	3′-UTR	128	0.45	C/T
INDEL10	10,066,364	3′-UTR	141	0.20	CA/C
SNP11	10,066,666	3′-UTR	113	0.18	G/A
INDEL12	10,066,708	3′-UTR	79	0.08	T/TC
INDEL13	10,066,711	3′-UTR	118	0.33	C/CA
INDEL14	10,066,728	3′-UTR	136	0.26	GGACCTGCTCTTCT/G
SNP15	10,066,750	3′-UTR	127	0.44	C/G
SNP16	10,066,770	3′-UTR	141	0.49	T/A
SNP17	10,066,775	3′-UTR	147	0.41	T/C
SNP18	10,066,885	3′-UTR	158	0.35	T/C
SNP19	10,066,886	3′-UTR	158	0.35	G/A
INDEL20	10,066,928	3′-UTR	157	0.36	CGA/C
SNP21	10,067,012	3′-UTR	156	0.34	A/G
SNP22	10,067,013	3′-UTR	155	0.31	C/T
SNP23	10,067,104	3′-UTR	159	0.03	G/A
INDEL24	10,067,277	3′-UTR	156	0.01	GC/G
SNP25	10,067,474	3′-UTR	144	0.50	A/G
SNP26	10,067,498	3′-UTR	153	0.38	C/A
SNP27	10,067,745	3′-UTR	152	0.28	T/G
SNP28	10,067,850	3′-UTR	158	0.02	G/A
SNP29	10,067,921	3′-UTR	157	0.28	A/G
SNP30	10,068,080	3′-UTR	156	0.31	T/C
SNP31	10,068,240	3′-UTR	156	0.35	A/G
SNP32	10,068,423	3′-UTR	116	0.41	T/C
INDEL33	10,068,438	3′-UTR	96	0.09	CA/C

^a^*n*: Number of animals genotyped.

^b^MAF: Minor allele frequency.

### Genotyping *DGAT2*

All pigs (*n* = 1129) used in the experiment were genotyped for SNP ss7315407085 in exon 9 of *DGAT2* using the primers described in Supplementary Table S1. Amplifications were performed by real-time PCR (QuantStudio3, Applied Biosystems, Thermo Scientific, Waltham, MA, USA) with High-Resolution Melt analysis (Luminaris Colour HRM Master Mix, Thermo Scientific) using 20 ng of genomic DNA and 0.4 µM of each primer in 5 µL final volume reaction. Thermocycling conditions were 50 °C 2 min, 95 °C 10 min, and 40 cycles of 95 °C 15 s, 60 °C 1 min, followed by a high-resolution melting curve starting with a denaturation at 95 °C for 15 s, annealing at 60 °C for 1 min and a slow ramp at 0.015 °C/s up to 95 °C. High Resolution Melt software v3.1 (Applied Biosystems, Thermo Scientific) was used for melting data analysis and sample genotyping. All pigs were also genotyped for the *SCD* (rs80912566 T > C; on SSC14) and leptin receptor (rs709596309 C > T; on SSC6) SNPs following the protocols described in Refs.^24,33^, respectively.

### *DGAT2* expression

*DGAT2* expression was measured by quantitative real-time PCR (qPCR) in the SM of 40 pigs from a single batch. RNA was isolated with TRI-Reagent according to the manufacturer’s protocol and purity was assessed by spectrophotometry with a Nanodrop-1000. Total RNA (1.5 µg) was reverse-transcribed using SuperScript IV Reverse Transcriptase (Invitrogen, Thermo Scientific) with 100 µM random hexamers at 23 °C for 10 min, 50 °C for 20 min and 80 °C for 10 min. The cDNA was diluted 1:30 in water. Real-time PCR assays were carried out in triplicate using SYBR Green Supermix (Bio-Rad, Hercules, CA, USA), 0.2 µM of each primer and 3 µl of diluted cDNA. The *B2M* and *RPL32* genes were used as reference genes to quantify and normalize the *DGAT2* expression data. Primers used for *DGAT2* expression (q*DGAT2*) are given in Supplementary Table S1.

### Estimation of *DGAT2* tag-SNP effects

The effect of the *DGAT2* ss7315407085 G > A SNP genotypes on production traits (body weight, carcass weight, BT and LT) and IMF content and fatty acid composition was estimated using a mixed model that included the batch (15 levels) and the genotype for *DGAT2* (GG, AG and AA), *SCD* (TT, CT and CC) and leptin receptor (TT, CT and CC) as fixed effects and the sire and the dam as random effects. The leptin receptor polymorphism was included in the model because of its impact on fat content and fatty acid composition^33^. The slaughter age, for production traits, and IMF content, for fatty acids, were added as covariates. The additive and dominant effects of the *DGAT2* SNP were estimated by replacing the genotype effect with the covariates (1, 0, − 1) and (0, 1, 0) for the GG, AG and AA genotypes, respectively. Gene expression was analysed only as a function of the *DGAT2* genotype. The effects of the genotype and covariates were tested using the F-statistic. Multiple pairwise comparisons among *DGAT2* genotypes were tested with the Tukey HSD test. Results are presented as least-square means ± standard error. All the analyses were performed using the statistical package JMP Pro 14 (SAS Institute Inc., Cary, NC).

### Consent for publication

All the authors read and agree to the content of this paper and its publication.

## Results

**Figure 2:**
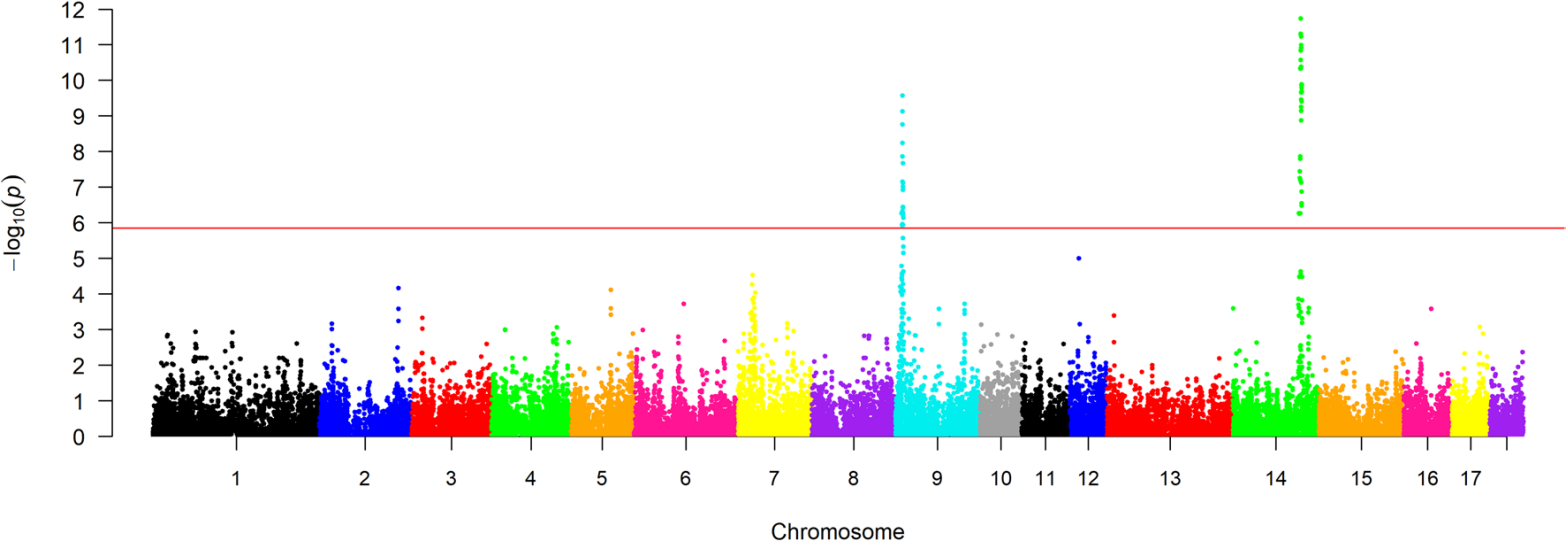
Genome-wide associations for palmitoleic acid content in the *gluteus medius* muscle. Different chromosomes are distinguished with different colours. The red line indicates the Bonferroni‐corrected genome‐wide significance threshold.

The preliminary GWAS revealed two regions associated with C16:1n-7, one on SSC9 and another on SSC14 (Fig. 2). The region on SSC9 (9.8 Mb to 12.8 Mb) contained 18 significant SNPs (P < 1.4 × 10^–6^). Candidate gene *DGAT2* mapped within this region, specifically at 10.0–10.1 Mb. This association was not identified in previous analyses in this Duroc line^32^ and evidence for potential implications of sequence variation in *DGAT2* on fat content and composition in pigs is still very scarce^14,21^. On the other hand, the region on SSC14 matched the variant in *SCD* described above^24^. No evidence of association was found between the genomic region of *DGAT1* (on SSC4) and fat content and fatty acid composition.

### Sequence variation in *DGAT2*

Using whole-genome sequencing data from pigs of the same line, we identified a total of 33 sequence variants in *DGAT2.* Three of them were exonic, although synonymous (Table 1) and, from these, only SNP5 in exon 9 (ss7315407085) had a minor allele frequency greater than 0.25 (0.33). With 1000 samples, which are around the current sample size of our biorepository, 0.25 is the minimum minor allele frequency required to detect (P < 0.05) a difference of around 5% (0.5 SD) between genotypes for C16:1n-7 with a power of at least 80%. This SNP has been previously reported in other pig breeds and crossbreds^14,21^ and, in line with our results, the A allele was the minor allele in all genetic types, with a frequency that ranged from 0 to 0.32. The other 30 variants were located in the promoter region (2 SNPs) and in the 3′-UTR (21 SNPs and 7 indels). Using only the sequenced pigs, we confirmed that *DGAT2* exerted an additive influence on C16:1n-7 (Supplementary Table S2), with SNP5 and the sequence variants in the interval between INDEL13 and SNP22 presenting the most relevant associations. Linkage disequilibrium of all variants was analysed with Haploview 4.2 software (Supplementary Fig. S1), showing that SNP5 was in linkage disequilibrium with both INDEL13 (r^2^ = 0.51) and SNP22 (r^2^ = 0.47). In view of these results, we selected SNP5 (ss7315407085) as a tag variant of this haplotype for further validation.

### Validation of *DGAT2* tag SNP

**Figure 3:**
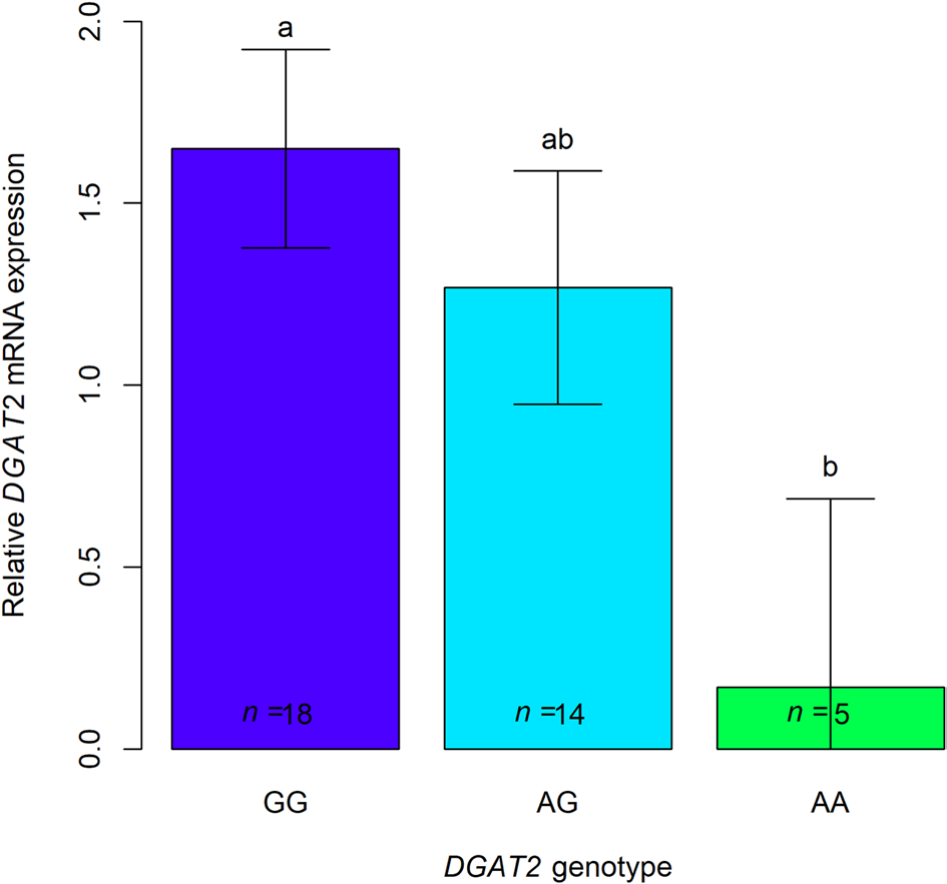
Relative expression of *DGAT2* across ss7315407085 genotypes. Numbers in boxes indicate the number of pigs used per genotype (*n*). Error bars represent standard errors. Columns lacking a common letter differ (P < 0.05).

The expression of *DGAT2* by ss7315407085 genotype is shown in Fig. 3. Despite the limited sample size, the results obtained indicate that the *DGAT2*-G show a favourable additive effect as compared to *DGAT2*-A on *DGAT2* mRNA expression in muscle (+ 0.65 ± 0.27, P < 0.05). The *DGAT2*-GG pigs displayed around 1.5-fold increase in *DGAT2* expression compared to the *DGAT2*-AA pigs (+ 1.48 ± 0.58, P < 0.05). Gene expression was measured in SM because it was the only muscle that could be sampled immediately after slaughter.

The effect of the *DGAT2* SNP on fatty acid composition was validated in GM (Table 2) and LM (Table 3) using the whole set of pigs in the experiment. In line with preliminary association results in Supplementary Table S2, the *DGAT2*-G allele exerted a positive additive effect on C16:1n-7 in both muscles (+ 0.12% ± 0.03, in GM, and + 0.19% ± 0.03, in LM, P < 0.0001). The effect of the *DGAT2*-G allele had also a positive impact on C14:0 (+ 0.04% ± 0.01, in GM, and + 0.05% ± 0.01, in LM, P < 0.01), C16:0 (+ 0.12% ± 0.06, in GM, P = 0.05) and C18:1n-7 (+ 0.06% ± 0.02, in GM, P = 0.01). However, the effect of the *DGAT2*-G allele was proportionally greater in C16:1n-7 than in C16:0, as evidenced by subsequent changes in the C16:1n-7 to C16:0 ratio (+ 0.04 ± 0.01, in GM, and + 0.07 ± 0.01, in LM, P < 0.01, values × 10). In contrast, the *DGAT2*-G allele affected negatively C18:0 and C18:1n-9, particularly in LM (− 0.13% ± 0.06, P < 0.05, and − 0.40% ± 0.15, P < 0.01, respectively).

**Table 2:** Least square means and additive values (± standard error) for fatty acids content and fatty acid ratios in the *gluteus medius* muscle by *DGAT2* genotype.

Trait^A^	*DGAT2* genotype			Additive value^B^	
	GG (*n* = 485)	AG (*n* = 482)	AA (*n* = 126)	a	P-value
**Fatty acid, %**					
C14:0	1.65 ± 0.01^a^	1.61 ± 0.01^b^	1.57 ± 0.02^b^	0.04 ± 0.01	**< 0.01**
C16:0	23.98 ± 0.06	23.94 ± 0.06	23.74 ± 0.11	0.12 ± 0.06	0.05
C18:0	11.35 ± 0.05^b^	11.51 ± 0.05^a^	11.51 ± 0.09^a,b^	− 0.08 ± 0.05	0.10
SFA	37.18 ± 0.10	37.27 ± 0.10	37.04 ± 0.18	0.07 ± 0.10	0.48
C16:1n-7	3.79 ± 0.03^a^	3.66 ± 0.03^b^	3.55 ± 0.05^c^	0.12 ± 0.03	**< 0.0001**
C18:1n-7	4.79 ± 0.04^a^	4.74 ± 0.03^a,b^	4.66 ± 0.05^b^	0.06 ± 0.02	**0.01**
C18:1n-9	41.01 ± 0.16	41.27 ± 0.14	41.25 ± .21	− 0.12 ± 0.11	0.29
MUFA	49.52 ± 0.10	49.57 ± 0.10	49.59 ± 0.18	− 0.03 ± 0.10	0.73
C18:2n-6	10.69 ± 0.07	10.62 ± 0.07	10.79 ± 0.12	− 0.06 ± 0.07	0.40
C18:3n-3	0.60 ± 0.005	0.60 ± 0.005	0.61 ± 0.01	− 0.01 ± 0.01	0.23
PUFA	13.29 ± 0.09	13.16 ± 0.09	13.36 ± 0.15	− 0.04 ± 0.08	0.66
**Fatty acid ratio**					
C16:1n-7/C16:0 (× 10)	1.59 ± 0.01^a^	1.54 ± 0.01^b^	1.51 ± 0.02^b^	0.04 ± 0.01	**< 0.01**
C16:1n-7/C18:1n-9 (× 10)	0.81 ± 0.01^c^	0.84 ± 0.01^b^	0.89 ± 0.02^a^	0.04 ± 0.01	**< 0.0001**
C18:1n-7/C18:0 (× 10)	4.27 ± 0.06^a^	4.13 ± 0.05^a,b^	4.02 ± 0.08^b^	0.13 ± 0.04	**< 0.01**
C18:1n-9/C18:0	3.66 ± 0.04	3.60 ± 0.03	3.55 ± 0.05	0.06 ± 0.03	0.06
(C16:1n-7 + C18:1n-7)/C16:0 (× 10)	3.39 ± 0.04^a^	3.30 ± 0.03^b^	3.21 ± 0.05^b^	0.09 ± 0.02	**< 0.01**

^A^SFA: saturated fatty acids (C14:0 + C16:0 + C18:0 + C20:0); MUFA: monounsaturated fatty acids (C16:1n-7 + C18:1n-9 + C18:1n-7 + C20:1n-9); and PUFA: polyunsaturated fatty acids (C18:2n-6 + C18:3n-3 + C20:2n-6 + C20:4n-6).

^B^Additive allele substitution of G for A.

^a,b,c^Within trait, means with different superscripts differ significantly (P < 0.05).

Bold font indicates statistical significance.

**Table 3:** Least square means and additive values (± standard errors) for fatty acids content and fatty acid ratios in *longissimus dorsi* by *DGAT2* genotype.

Trait^A^	*DGAT2* genotype			Additive value^B^	
	GG (*n* = 218)	AG (*n* = 238)	AA (*n* = 70)	a	P-value
**Fatty acid, %**					
C14:0	1.63 ± 0.02^a^	1.57 ± 0.01^b^	1.52 ± 0.02^b^	0.05 ± 0.01	**< 0.0001**
C16:0	25.07 ± 0.08	25.03 ± 0.08	24.98 ± 0.12	0.05 ± 0.07	0.46
C18:0	12.13 ± 0.07	12.25 ± 0.06	12.38 ± 0.11	− 0.13 ± 0.06	**0.04**
SFA	39.02 ± 0.13	39.04 ± 0.13	39.08 ± 0.2	− 0.03 ± 0.12	0.80
C16:1n-7	4.04 ± 0.04^a^	3.88 ± 0.04^b^	3.67 ± 0.06^c^	0.19 ± 0.03	**< 0.0001**
C18:1n-7	4.76 ± 0.04	4.76 ± 0.04	4.67 ± 0.06	0.05 ± 0.03	0.10
C18:1n-9	41.10 ± 0.22^b^	41.69 ± 0.20^a^	41.87 ± 0.29^a^	− 0.40 ± 0.15	**< 0.01**
MUFA	50.29 ± 0.14	50.38 ± 0.13	50.33 ± 0.21	− 0.02 ± 0.12	0.84
C18:2n-6	8.32 ± 0.07	8.26 ± 0.07	8.29 ± 0.11	0.02 ± 0.06	0.78
C18:3n-3	0.37 ± 0.004	0.38 ± 0.004	0.38 ± 0.01	− 0.00 ± 0.00	0.29
PUFA	10.69 ± 0.09	10.58 ± 0.09	10.60 ± 0.15	0.05 ± 0.08	0.57
**Fatty acid ratio**					
C16:1n-7/C16:0 (× 10)	1.62 ± 0.01^a^	1.55 ± 0.01^b^	1.47 ± 0.02^c^	0.07 ± 0.01	**< 0.0001**
C16:1n-7/C18:1n-9 (× 10)	0.86 ± 0.02^c^	0.91 ± 0.02^b^	0.96 ± 0.02^a^	0.05 ± 0.01	**< 0.0001**
C18:1n-7/C18:0 (× 10)	4.06 ± 0.07	4.03 ± 0.06	3.91 ± 0.09	0.07 ± 0.05	0.17
C18:1n-9/C18:0	3.47 ± 0.05	3.51 ± 0.04	3.48 ± 0.06	− 0.00 ± 0.04	0.93
(C16:1n-7 + C18:1n-7)/C16:0 (× 10)	3.40 ± 0.04	3.36 ± 0.04	3.27 ± 0.06	0.07 ± 0.03	**0.03**

^A^SFA: saturated fatty acids (C14:0 + C16:0 + C18:0 + C20:0); MUFA: monounsaturated fatty acids (C16:1n-7 + C18:1n-9 + C18:1n-7 + C20:1n-9); and PUFA: polyunsaturated fatty acids (C18:2n-6 + C18:3n-3 + C20:2n-6 + C20:4n-6).

^B^Additive allele substitution of G for A.

^a,b,c^ Within trait, means with different superscripts differ significantly (P < 0.05).

Bold font indicates statistical significance.

**Figure 4:**
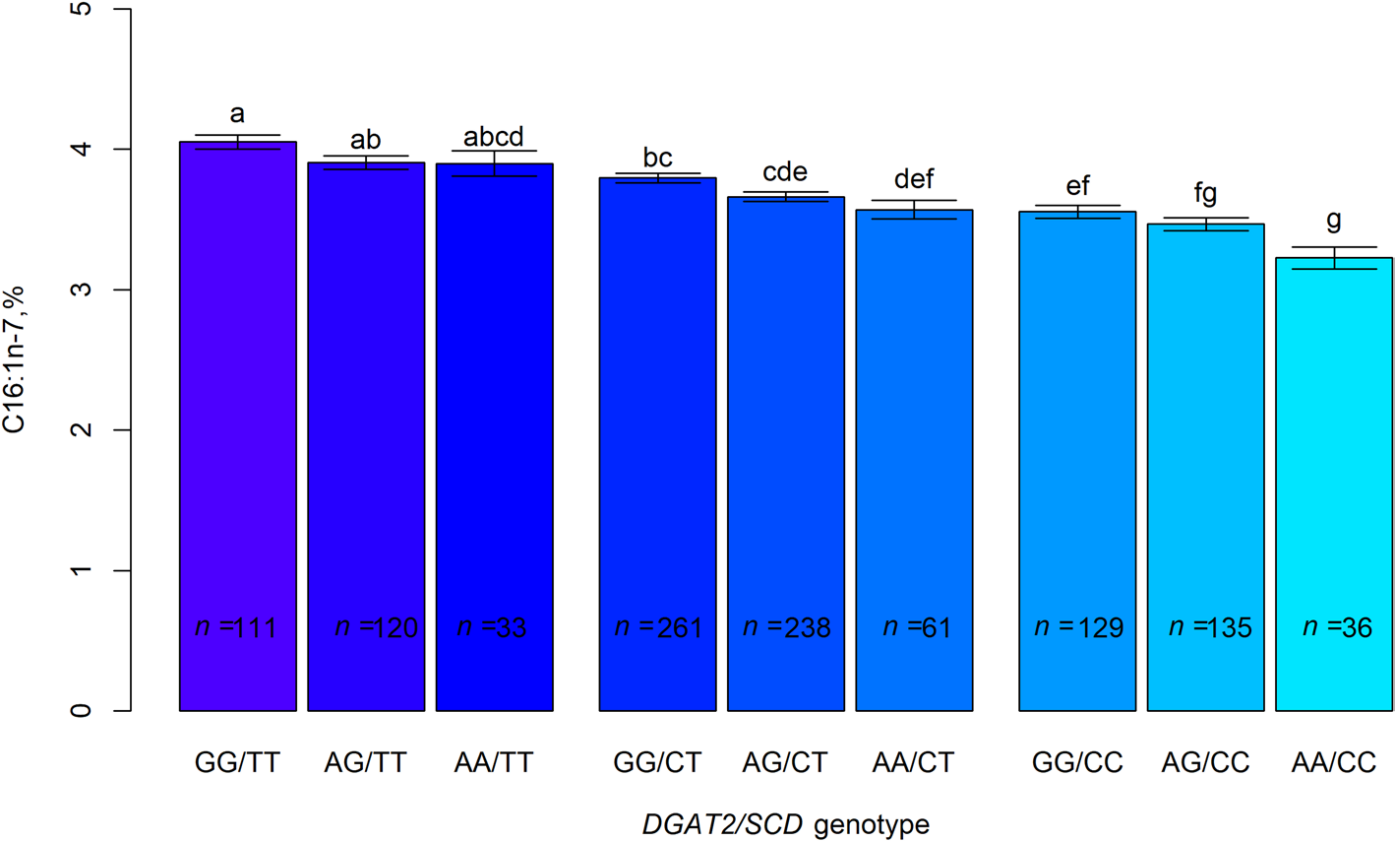
Effect of *DGAT2* by *SCD* genotype on palmitoleic acid (C16:1n-7) in muscle *gluteus medius*. Numbers in boxes indicate the number of pigs used per genotype (*n*). Error bars represent standard errors. Columns lacking a common letter differ (P < 0.05).

The *DGAT2* SNP did not show dominance effects nor evidence for interaction with the *SCD* genotype for any of the fatty acids. The additive behaviour of the *DGAT2*-G allele was mantained across all *SCD* genotypes (Fig. 4). Thus, the maximum difference in C16:1n-7, which accounted for around 25% of the mean, occurred between the two extreme *DGAT2*/*SCD* haplotypes (from 4.05% ± 0.05, for GG/TT, to 3.23% ± 0.08, for AA/CC, P < 0.0001). Despite this, the difference between *DGAT2*-GG and AA genotypes was greater for the *SCD*-CC genotype (+ 0.32% ± 0.09, P < 0.001) than for the *SCD*-TT genotype (+ 0.15% ± 0.10, P = 0.13).

We did not find evidence of consistent effects of *DGAT2* on body weight or carcass fat content (Table 4) or on the fatty acid composition of SF (Supplementary Table S3) or liver (Supplementary Table S4). Only a minor effect on live body weight was observed (Table 4). Although *DGAT2*-GG pigs weighted 2.0 kg ± 0.7 (P < 0.05) less than *DGAT2*-AG pigs, the G allele did not show a clear additive behaviour (− 1.0 kg ± 0.6, P = 0.08).

**Table 4:** Least square means and additive values (± standard error) for body weight (BW), carcass weight (CW), backfat (BT) and loin (LT) thickness and intramuscular fat (IMF) content by *DGAT2* genotype.

Trait	*DGAT2* genotype			Additive value^A^	
	GG (*n* = 496)	AG (*n* = 488)	AA (*n* = 129)	a	P-value
**Live measurements**					
BW, kg	124.3 ± 0.6^b^	126.3 ± 0.6^a^	126.4 ± 1.1^a,b^	− 1.0 ± 0.6	0.08
BT, mm	21.9 ± 0.2	22.1 ± 0.2	22.4 ± 0.4	− 0.3 ± 0.2	0.20
LT, mm	47.5 ± 0.3	47.1 ± 0.3	47.7 ± 0.5	− 0.1 ± 0.3	0.83
**Carcass measurements**					
CW, kg	96.0 ± 0.5	97.4 ± 0.5	97.1 ± 0.9	− 0.6 ± 0.5	0.24
BT, mm	25.6 ± 0.2	26.0 ± 0.2	26.4 ± 0.4	− 0.4 ± 0.2	0.08
LT, mm	44.1 ± 0.4	43.8 ± 0.4	43.8 ± 0.7	0.1 ± 0.4	0.74
**IMF content, % dry matter**					
*M. gluteus medius*	18.1 ± 0.3	17.6 ± 0.3	18.1 ± 0.5	− 0.02 ± 0.03	0.94
*M. longissimus thoracis*	13.3 ± 0.3	13.5 ± 0.3	13.0 ± 0.4	0.16 ± 0.23	0.48

^A^Additive allele substitution of G for A.

^a,b^Within trait, means with different superscripts differ significantly (P < 0.05).

## Discussion

Understanding the regulation of fat metabolism is a milestone in the prevention and treatment of human lipid diseases, such as obesity, and in animal science. In this context, DGAT2 has received attention as the enzyme that catalyzes the final step in the synthesis of TG, the most common type of body fat. Here, we validated the effects of the ss7315407085 G > A SNP on fat content and fatty acid composition as a tag SNP for the haplotype in *DGAT2* that was identified following a preliminary GWAS and after mRNA expression and sequence variant association analyses. Using a larger sample, we confirmed that the minor allele A segregates in the studied Duroc line at a moderate frequency (0.33), and that it has a negative impact on C16:1n-7 content in muscle. The moderate minor allele frequency in the studied Duroc line is similar to estimates from other Duroc lines^14^ and White and Asian breeds and crossbreds^14,21,22^.

Our validation scheme evidenced a consistent effect of *DGAT2* SNP on C16:1n-7 in muscle that is independent of IMF content. A genetic variant in *SCD* with relevant effects on MUFA also segregates in this line^24^. The *DGAT2* SNP had a lower impact on C16:1n-7 than the *SCD* SNP variant, particularly in terms of genetic variance. While the additive value of *DGAT2* for C16:1n-7 was around half of that of *SCD*, the additive variance only reached 22% of that attributed to *SCD*. It has been shown that *SCD* co-expresses with *DGAT2*^34^ and that their respective enzymes locate very close in the endoplasmic reticulum^11,17^, two findings that support the hypothesis that SCD is involved in the TG synthesis by providing a more accessible pool of MUFA through substrate channelling^12,17^. This also would explain why cells overexpressing *DGAT2* show a greater proportion of MUFA and very particularly of C16:1n-7^34^. Our results are in line with this hypothesis, because C16:1n-7 was the fatty acid most affected by the *DGAT2* SNP and the G allele, the one with higher *DGAT2* expression, was the one that led to accumulate more C16:1n-7. However, we did not find evidence for a positive interaction between *SCD* and *DGAT2* SNPs for C16:1n-7. Using RNAseq data from 40 other pigs of the same line, we were able to confirm that *DGAT2* genotype (GG: n = 19, AG: n = 14, AA: n = 5) affected muscular *DGAT2* expression, but we did not detect differential expression of *SCD* by *DGAT2* genotype. In absence of interaction, the two genes mostly behaved in an allele-dose manner (Fig. 3).

The positive effect of the *DGAT2* G allele on C16:1n-7 was not observed on C18:1n-9. Zhang et al.^34^ evidenced that the impact of DGAT2 on C16:1n-7 was proportionally greater as fatty acid composition turned more dependent on de novo fatty acids. For C18:1n-9, the same effect was only seen in mature adipocytes, especially when they were cultured in a fatty acid-rich medium. In line with these results, a plausible explanation to the differential trend between C16:1n-7 and C18:1n-9 could be that the most immediate (short-term) effect of DGAT2 is to alter fatty acid composition by selectively capturing as substrate the first available de novo fatty acids rather than to enhance the endogenous biosynthesis of longer-chain fatty acids. This may be the case here, since differences in *DGAT2* expression across genotypes were relatively small and no correlated responses in IMF content and *SCD* expression were observed. The favourable effect of the G allele on C14 and C16 fatty acids would reinforce this hypothesis. On the other hand, the impact of de novo fatty acids on final fatty acid composition is more apparent in C16:1n-7 and C18:1n-7 than in C18:1n-9, which is proportionally more abundant in pig feed than C16:1n-7 and thus more likely to be influenced by the diet. Similarly, the effect of *DGAT2* on fatty acid composition was only observed in IMF, which is precisely the adipose tissue that develops later^35^ and is less sensitive to dietary fat^36^.

Changes in *DGAT2* expression are related to lipid accumulation. Results in human and murine cells indicate that DGAT2 promotes TG synthesis and storage in cytosolic lipid droplets^37^ and accordingly *DGAT2* is expressed more abundantly in cells with greater de novo fatty acid synthesis^19^. This is in line with findings in pigs, in which *DGAT2* was most expressed in the liver of fatter breeds, where TG are expected to be more actively synthesized^38^. The expression of *DGAT2* was twofold higher in fatter than in leaner breeds and around fivefold and tenfold higher in liver than in subcutaneous fat and muscle, respectively. However, the mRNA expression of *DGAT2* was positively correlated with IMF content but not with BT. In view of these results, it would appear that the pigs carrying the *DGAT2* A allele, which down-regulates the expression of *DGAT2*, should be less fatty and, specifically, have less IMF content. Even though this association was reported in other studies^14,21^, we did not find evidence that *DGAT2* A entails a reduction in IMF content, BT or even fat content in liver. This may contradict biological expectations, but, in comparison with previous reports, validation here was performed using a much larger set of pigs from a single line so as to avoid breed or family biases. A 13-bp deletion in *DGAT2* 3′-UTR region has also been associated with increased *DGAT2* mRNA expression and BT^39^. This deletion allele showed higher transcriptional activity, most likely owing to a less stable 3′-UTR secondary structure. This indel was located at 905 bp downstream from the stop codon and matches INDEL14 (Table 1). In line with the results observed for SNP5, none of these polymorphisms including INDEL14 (r^2^ = 0.57, Supplementary Fig. S1) affected BT in our Duroc line (Supplementary Table S2).

The results obtained are more appealing for lipid research than for pig breeding. In recent years, C16:1n-7 has received a lot of attention for its potential role as a lipokine, i.e. as a lipid hormone that acts in distant organs. Despite this, the effects of C16:1n-7 are still under debate. On the one hand, preclinical experiments with cell and rodent models show that C16:1n-7 supplementation has anti-inflammatory properties^40,41^ that protect against metabolic disorders. But, on the other hand, research in humans reported elevated blood levels of C16:1n-7 in patients with obesity and metabolic syndrome^42,43^. Findings in pigs may contribute to disentangle the biological implications of C16:1n-7 as well as to understand the genetic regulation of fat metabolism.

## Conclusions

A sequence variant in the porcine *DGAT2* influences muscular gene expression and fatty acid composition, with changes mostly limited to swapping C18 fatty acids for C14 and C16 fatty acids of the same degree of saturation. Differences across *DGAT2* genotypes are quantitatively small, but the variant that enhances *DGAT2* expression is associated with increased content of shorter-chain fatty acids, thereby indicating that DGAT2 preferentially uptakes the first available de novo fatty acids as substrate. Although *DGAT2* and *SCD* act additively, the fact that *DGAT2* overexpression impacts C16:1n-7 more than C16:0 indicates that *DGAT2* and *SCD* are closely interlinked. Further enzyme characterization is needed, but our findings provide evidence that C16:1n-7 is the immediate substrate for DGAT2 and corroborate that fatty acid metabolism in pigs is subjected to subtle tissue-specific genetic regulatory mechanisms.

## Supplementary Information

Supplementary Information.

## Data Availability

Data that support the findings of this study are available within the article and Supplementary Information, or from the authors upon reasonable request.

## Abbreviations

BT: Backfat thickness
DGAT: Diacylglycerol *O*-acyltransferase
GM: *G**luteus medius* muscle
GWAS: Genome-wide association study
IMF: Intramuscular fat
LM: *Longissimus thoracis* muscle
LT: Loin thickness
MUFA: Monounsaturated fatty acids
PUFA: Polyunsaturated fatty acids
SCD: Stearoyl-CoA desaturase
SD: Standard deviation
SF: Subcutaneous fat
SFA: Saturated fatty acids
SM: *Semimembranosus* muscle
SNP: Single nucleotide polymorphism
TG: Triacylglycerols
