# Supplementary material for: A sequence variant in the diacylglycerol O-acyltransferase 2 gene influences palmitoleic acid content in pig muscle
Source: Sci Rep. 2021 Jul 20;11:14797. doi: 10.1038/s41598-021-94235-z (PMC8292425; doi:10.1038/s41598-021-94235-z)
Supplement: Supplementary file 1 — Supplementary Information. [file 41598_2021_94235_MOESM1_ESM.docx]

**Supplementary Information**

**A sequence variant in the diacylglycerol O-acyltransferase 2 gene influences palmitoleic acid content in pig muscle**

Emma Solé, Roger Ros-Freixedes, Mar Tor, Ramona N. Pena and Joan Estany

Department of Animal Science, University of Lleida-Agrotecnio-CERCA Center, 25198 Lleida, Catalonia, Spain.

**Table S1.** Primers used for genotyping the polymorphism in exon 9 of the *DGAT2* gene and primers used to analyse its gene expression

| **Primer** | **Sequence 5’ 🡪 3’** | **Tm** | **PCR size** | **Protocol** |
| --- | --- | --- | --- | --- |
| *DGAT2* Fw | TCAACCAGCACAAGACCAAG | 59.9 | 58bp | Genotyping |
| *DGAT2* Rv | CAGTTCACCTCCAGGACCTC | 59.7 |  |  |
| *qDGAT2* Fw | AGGACATTGACCTCTACCATGC | 60.4 | 109bp | Gene expression |
| *qDGAT2* Rv | CAGTTCACCTCCAGGACCTC | 59.7 |  |  |

**Table S2**. Additive values (± standard errors) of sequence variants in the genomic region that harbours the *DGAT2* gene (Table 1) and associated P-values for palmitoleic acid (C16:1n7, % of fatty acids) and intramuscular fat (IMF, in % dry matter) in muscle *gluteus medius* and backfat thickness (BT) using data from 199 sequenced pigs.

|  |  | C16:1n-7, % | |  | | IMF, % | |  | BT, mm | |
| --- | --- | --- | --- | --- | --- | --- | --- | --- | --- | --- |
| Variant |  | a | P-value |  | a | | P-value |  | a | P-value |
| SNP1 |  | -0.12±0.10 | 0.23 |  | -0.77±1.08 | | 0.48 |  | 1.66±1.60 | 0.31 |
| SNP2 |  | 0.32±0.48 | 0.51 |  | 9.65±4.26 | | 0.03 |  | 10.19±5.84 | 0.09 |
| SNP3 |  | -0.06±0.09 | 0.49 |  | -0.91±0.81 | | 0.26 |  | 0.01±1.03 | 0.99 |
| SNP4 |  | 0.03±.0.09 | 0.77 |  | -1.20±0.87 | | 0.17 |  | -1.15±1.10 | 0.30 |
| SNP5 |  | 0.14±0.06 | **0.03** |  | -0.17±0.53 | | 0.76 |  | -0.12±0.78 | 0.88 |
| SNP6 |  | 0.10±0.07 | 0.12 |  | -0.57±0.57 | | 0.32 |  | -0.74±0.82 | 0.37 |
| SNP7 |  | -0.11±0.05 | **0.02** |  | -0.56±0.42 | | 0.18 |  | 0.09±0.53 | 0.87 |
| SNP8 |  | 0.04±0.05 | 0.46 |  | 0.82±0.46 | | 0.08 |  | 0.70±0.56 | 0.22 |
| SNP9 |  | 0.08±0.05 | 0.07 |  | 0.73±0.45 | | 0.11 |  | 0.55±0.58 | 0.35 |
| INDEL10 |  | 0.09±0.06 | 0.13 |  | -0.20±0.50 | | 0.69 |  | -0.32±0.62 | 0.60 |
| SNP11 |  | 0.11±0.06 | 0.09 |  | 0.14±0.57 | | 0.81 |  | -0.11±0.70 | 0.88 |
| INDEL12 |  | -0.08±0.15 | 0.59 |  | 2.24±1.49 | | 0.14 |  | 3.05±2.06 | 0.14 |
| INDEL13 |  | 0.12±0.05 | **0.01** |  | -0.72±0.45 | | 0.11 |  | 0.06±0.61 | 0.92 |
| INDEL14 |  | 0.13±0.05 | **<0.01** |  | -0.44±0.46 | | 0.34 |  | 0.11±0.58 | 0.85 |
| SNP15 |  | 0.10±0.04 | **0.02** |  | -0.35±0.43 | | 0.43 |  | 0.53±0.57 | 0.36 |
| SNP16 |  | 0.10±0.04 | **0.02** |  | -0.03±0.41 | | 0.95 |  | 0.11±0.51 | 0.82 |
| SNP17 |  | -0.11±0.04 | **<0.01** |  | -0.03±0.43 | | 0.95 |  | -0.22±0.53 | 0.67 |
| SNP18 |  | -0.11±0.05 | **0.03** |  | 0.05±0.49 | | 0.92 |  | -0.23±0.59 | 0.70 |
| SNP19 |  | -0.11±0.06 | **0.03** |  | 0.05±0.49 | | 0.92 |  | -0.23±0.59 | 0.70 |
| INDEL20 |  | -0.10±0.05 | 0.06 |  | -0.11±0.47 | | 0.82 |  | -0.24±0.58 | 0.69 |
| SNP21 |  | -0.14±0.05 | **<0.01** |  | -0.34±0.50 | | 0.49 |  | 0.05±0.59 | 0.94 |
| SNP22 |  | 0.15±0.05 | **<0.01** |  | 0.15±0.45 | | 0.75 |  | -0.83±0.54 | 0.13 |
| SNP23 |  | 0.23±0.15 | 0.12 |  | 1.42±1.37 | | 0.30 |  | 2.92±1.67 | 0.08 |
| INDEL24 |  | 0.12±0.32 | 0.72 |  | -0.73±3.00 | | 0.81 |  | -3.88±3.66 | 0.29 |
| SNP25 |  | 0.12±0.32 | 0.72 |  | 0.04±0.43 | | 0.92 |  | -0.44±0.54 | 0.41 |
| SNP26 |  | -0.06±0.05 | 0.24 |  | -0.37±0.46 | | 0.42 |  | -0.34±0.57 | 0.55 |
| SNP27 |  | -0.02±0.05 | 0.64 |  | 0.08±0.49 | | 0.87 |  | 1.10±0.60 | 0.07 |
| SNP28 |  | -0.02±0.16 | 0.92 |  | -0.92±1.47 | | 0.53 |  | -0.11±1.79 | 0.95 |
| SNP29 |  | -0.04±0.05 | 0.50 |  | -0.25±0.50 | | 0.61 |  | 0.99±0.60 | 0.10 |
| SNP30 |  | -0.09±0.05 | 0.10 |  | -0.25±0.51 | | 0.62 |  | 0.06±0.62 | 0.92 |
| SNP31 |  | 0.00±0.05 | 0.97 |  | 0.22±0.46 | | 0.64 |  | 1.09±0.56 | 0.05 |
| SNP32 |  | 0.05±0.05 | 0.30 |  | 0.06±0.48 | | 0.91 |  | 1.26±0.60 | 0.04 |
| INDEL33 |  | 0.10±0.10 | 0.31 |  | -0.44±0.85 | | 0.61 |  | 0.52±1.02 | 0.61 |

Bold font indicates statistical significance.

**Table S3**. Least square means and additive values (± standard error) for fatty acids and ratios in subcutaneus fat by *DGAT2* genotype.

| Trait^1^ | *DGAT2* genotype | | |  | Additive value^2^ | |
| --- | --- | --- | --- | --- | --- | --- |
|  | GG (*n*=114) | AG (*n*=91) | AA (*n*=21) |  | a | P-value |
| Fatty acid, % |  |  |  |  |  |  |
| C14:0 | 1.46 ± 0.02 | 1.45 ± 0.02 | 1.41 ± 0.03 |  | 0.02 ± 0.01 | 0.14 |
| C16:0 | 22.02 ± 0.28 | 22.16 ± 0.31 | 21.51 ± 0.43 |  | 0.26 ± 0.20 | 0.20 |
| C18:0 | 11.23 ± 0.18 | 11.43 ± 0.20 | 11.14 ± 0.28 |  | 0.04 ± 0.13 | 0.73 |
| SFA | 34.90 ± 0.43 | 35.21 ± 0.48 | 34.44 ± 0.66 |  | 0.23 ± 0.30 | 0.46 |
| C16:1n-7 | 2.21 ± 0.05 | 2.19 ± 0.06 | 2.21 ± 0.08 |  | -0.00 ± 0.04 | 0.99 |
| C18:1n-7 | 5.28 ± 0.13 | 5.40 ± 0.10 | 5.45 ± 0.25 |  | -0.08 ± 0.14 | 0.54 |
| C18:1n-9 | 37.28 ± 0.4^b^ | 38.39 ± 0.30^a^ | 37.36 ± 0.81^ab^ |  | -0.04 ± 0.43 | 0.92 |
| MUFA | 47.05 ± 0.40 | 47.15 ± 0.42 | 47.65 ± 0.58 |  | -0.30 ± 0.27 | 0.26 |
| C18:2n-6 | 15.85 ± 0.30 | 15.37 ± 0.33 | 15.81 ± 0.46 |  | 0.02 ± 0.21 | 0.92 |
| C18:3n-3 | 1.10 ± 0.04 | 1.11 ± 0.04 | 1.11 ± 0.06 |  | -0.01 ± 0.03 | 0.74 |
| PUFA | 18.10 ± 0.34 | 17.61 ± 0.37 | 18.06 ± 0.52 |  | 0.02 ± 0.24 | 0.94 |
| Fatty acid ratio |  |  |  |  |  |  |
| C16:1n-7/C16:0 (x10) | 1.05 ± 0.03 | 1.02 ± 0.04 | 1.05 ± 0.05 |  | -0.00 ± 0.03 | 0.93 |
| C16:1n-7/C18:1n-9 (x10) | 0.54 ± 0.03 | 0.54 ± 0.03 | 0.56 ± 0.05 |  | -0.00 ± 0.02 | 0.90 |
| C18:1n-7/C18:0 (x10) | 4.54 ± 0.19 | 4.65 ± 0.16 | 4.78 ± 0.36 |  | -0.13 ± 0.19 | 0.53 |
| C18:1n-9/C18:0 | 3.19 ± 0.09 | 3.28 ± 0.07 | 3.29 ± 0.16 |  | -0.05 ± 0.09 | 0.58 |
| (C16:1n-7+C18:1n-7)/C16:0 (x10) | 3.30 ± 0.09 | 3.36 ± 0.08 | 3.45 ± 0.18 |  | -0.07 ± 0.09 | 0.44 |

^1^ SFA: saturated fatty acids (C14:0+C16:0+C18:0+C20:0); MUFA: monounsaturated fatty acids (C16:1n-9+C18:1n-9+C18:1n-7+C20:1n-9); and PUFA: polyunsaturated fatty acids (C18:2n-6+C18:3n-3+C20:2n-6+C20:4n-6).

^2^ Additive allele substitution of G for A

^a,b^  Within trait, means with different superscripts differ significantly (P<0.05).

**Table S4**. Least square means and additive values (± standard error) for fatty acids and ratios in liver by *DGAT2* genotype

| Trait^1^ | *DGAT2* genotype | | |  | Additive value^2^ | |
| --- | --- | --- | --- | --- | --- | --- |
|  | GG (*n*=76) | AG (*n*=65) | AA (*n*=16) |  | a | P-value |
| Fat content, %DM | 13.8 ± 0.3 | 14.0 ± 0.3 | 14.1 ± 0.6 |  | -0.16 ± 0.31 | 0.61 |
| Fatty acid, % |  |  |  |  |  |  |
| C14:0 | 0.5 ± 0.03 | 0.55 ± 0.03 | 0.46 ± 0.06 |  | 0.02 ± 0.03 | 0.59 |
| C16:0 | 17.60 ± 0.39 | 17.41 ± 0.38 | 16.98 ± 0.67 |  | 0.31 ± 0.35 | 0.37 |
| C18:0 | 25.83 ± 0.6 | 24.46 ± 0.58 | 25.59 ± 0.96 |  | 0.12 ± 0.48 | 0.8 |
| SFA | 45.93 ± 0.73 | 44.39 ± 0.71 | 44.87 ± 1.13 |  | 0.53 ± 0.56 | 0.34 |
| C16:1n-7 | 0.97 ± 0.04 | 0.99 ± 0.04 | 0.92 ± 0.07 |  | 0.03 ± 0.04 | 0.42 |
| C18:1n-7 | 2.65 ± 0.08 | 2.64 ± 0.07 | 2.53 ± 0.14 |  | 0.06 ± 0.07 | 0.41 |
| C18:1n-9 | 17.98 ± 0.64 | 18.03 ± 0.59 | 17.07 ± 1.08 |  | 0.46 ± 0.54 | 0.41 |
| MUFA | 21.5 ± 0.54 | 21.99 ± 0.53 | 20.86 ± 0.92 |  | 0.32 ± 0.47 | 0.49 |
| C18:2n-6 | 18.42 ± 0.26 | 18.74 ± 0.25 | 18.88 ± 0.42 |  | -0.23 ± 0.22 | 0.28 |
| C18:3n-3 | 0.54 ± 0.02 | 0.55 ± 0.02 | 0.55 ± 0.03 |  | -0.01 ± 0.02 | 0.74 |
| PUFA | 32.71 ± 0.95 | 33.69 ± 0.91 | 34.73 ± 1.55 |  | -1.01 ± 0.79 | 0.2 |
| Fatty acid ratio |  |  |  |  |  |  |
| C16:1n-7/C16:0 (x10) | 0.52 ± 0.02 | 0.54 ± 0.02 | 0.51 ± 0.03 |  | 0.01 ± 0.01 | 0.64 |
| C16:1n-7/C18:1n-9 (x10) | 0.57 ± 0.02 | 0.56 ± 0.02 | 0.53 ± 0.03 |  | 0.02 ± 0.01 | 0.15 |
| C18:1n-7/C18:0 (x10) | 1.11 ± 0.05 | 1.15 ± 0.05 | 1.04 ± 0.08 |  | 0.03 ± 0.04 | 0.46 |
| C18:1n-9/C18:0 | 0.75 ± 0.04 | 0.78 ± 0.04 | 0.69 ± 0.07 |  | 0.03 ± 0.04 | 0.41 |
| (C16:1n-7+ C18:1n-7)/C16:0 (x10) | 1.97 ± 0.04 | 2.02 ± 0.04 | 1.96 ± 0.07 |  | 0.00 ± 0.04 | 0.90 |

^1^ SFA: saturated fatty acids (C14:0+C16:0+C18:0+C20:0); MUFA: monounsaturated fatty acids (C16:1n-9+C18:1n-9+C18:1n-7+C20:1n-9); and PUFA: polyunsaturated fatty acids (C18:2n-6+C18:3n-3+C20:2n-6+C20:4n-6).

^2^ Additive allele substitution of G for A

^a,b^  Within trait, means with different superscripts differ significantly (P<0.05).


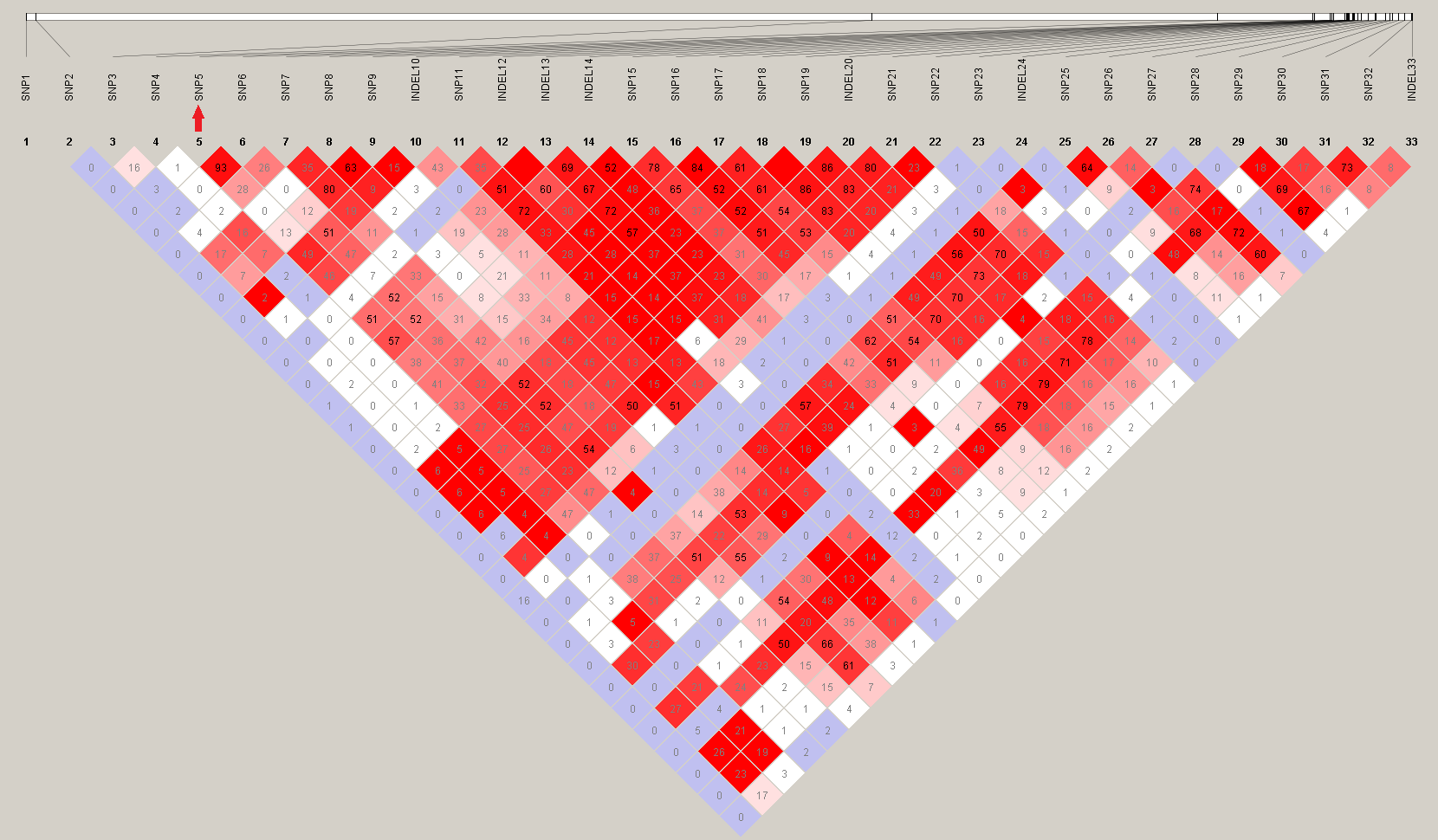
**Figure S1**. Linkage disequilibrium structure for the *DGAT2* gene. Featured variants in Table 1 are given above the plot and the red arrow indicates the location of the tag SNP (ss7315407085) used for further validations. Pairwise linkage disequilibrium coefficients (r^2^) are shown in each cell using the standard Haploview linkage disequilibrium colour scheme.
